# Characterization of gut microbiota in children with pulmonary tuberculosis

**DOI:** 10.1186/s12887-019-1782-2

**Published:** 2019-11-18

**Authors:** Weiran Li, Yu Zhu, Qiong Liao, Zhiling Wang, Chaomin Wan

**Affiliations:** 1 Department of Pediatrics, West China Second University Hospital, Sichuan University, No 20, 3rd section of Renmin South Road, Chengdu, 610041 People’s Republic of China; 2 Key Laboratory of Birth Defects and Related Diseases of Women and Children (Sichuan University), Ministry of Education, Chengdu, People’s Republic of China

**Keywords:** Pulmonary tuberculosis, Gut microbiota, 16SrDNA, Children

## Abstract

**Background:**

Gut microbiota plays a critical role in many important physiological processes and is linked with various pulmonary infectious diseases. The relationship between pulmonary tuberculosis (PTB) and gut microbiota has been poorly studied. The present study aimed to characterize gut microbiota in pediatric patients with PTB.

**Methods:**

A case-controlled study was executed for the characterization of gut microbiota in pediatric PTB patients. Fecal samples were collected from the PTB patients and healthy controls upon admission. In addition, a one-month follow-up assessment was performed to investigate alterations in the gut microbiota post anti-tuberculosis treatment. 16SrDNA sequencing analysis of fecal DNA was completed on the Illumina MiSeq platform.

**Results:**

Compared with healthy controls, the gut microbiota of pediatric patients with PTB was characterized by decreased microbial diversity. PTB patients further presented an up-regulation of the pro-inflammatory bacteria *Prevotella,* the opportunistic pathogen *Enterococcus,* as well as a reduction of beneficial bacteria including *Ruminococcaceae*, *Bifidobacteriaceae* and *prausnitzii.* One-month after anti-tuberculosis therapy, the richness of gut microbiota in PTB patients was distinctly depleted.

**Conclusions:**

The gut microbiota of pediatric patients with PTB was significantly distinct from healthy controls. Additionally, the richness of gut microbiota in PTB patients decreased after one-month anti-tuberculosis treatment. It is hypothesized that the homeostasis of gut microbiota in PTB patients may affect the pathogenies of PTB by de-regulation of the hosts’ immune status through the gut-lung axis.

## Background

Tuberculosis (TB) is one of the most common infectious disease in the world and the leading cause of death from a single infectious agent. TB is caused by *Mycobacterium tuberculosis*, a bacterium which attacks the lungs predominantly but can affect other organs as well. According to a 2018 WHO report, there were approximately 10.0 million people acquiring TB in 2017. Further, TB caused an estimated 1.3 million deaths in 2017. In addition to the high rate of TB incidence, a growing concern is the prevalence of drug-resistant TB. In 2017, 558,000 new TB patients exhibited resistance to the first-line drug rifampicin, more than 80% of which demonstrated eventual multidrug-resistant TB [1]. Thus, TB remains a significant public health problem requiring effective interventions.

With the development of next-generation high-throughput sequencing, the investigation of the human gut microbiome has become more feasible and has led to insights into gut microbiota. Gut microbiota are a complex and dynamic ecosystem harbouring more than 100 trillion commensal microorganisms, surpassing the number of human cells [2]. A great amount of evidence suggests that the gut microbiota exerts many beneficial effects on humans through the involvement physiological processes including digestion and absorption of nutrition, modulation of the immune system, and protection against pathogenic invasion [3–7]. Increasing lines of evidence have further indicated that the gut microbiota are linked to some systemic infectious diseases [8–10] such as HIV infection, chronic hepatitis C and bloodstream infection. In addition, the critical role of gut microbiota in pulmonary infectious disease has also been increasingly recognized and a vital cross-talk mechanism between the gut and lung (termed the “gut-lung axis”) has been previously proposed [11, 12]. The gut-lung axis is bidirectional and its interactions include two aspects: on the one hand, some respiratory diseases including asthma and influenza are strongly linked to a dysbiotic gut microbiota [13, 14]. On the other hand, a protective role of the gut microbiota against invasion of the lung by pathogenic microorganisms may be achieved by modulation of the local and systemic immunity of the host [7, 15, 16].

Although gut microbiota may impact the etiology of pulmonary diseases, there have been few explorations to-date focusing on the characterization of gut microbiota in TB patients, especially in pediatric groups which represent a clinically important population with increased susceptibility to TB [17]. Thus, the present study was developed to explore the gut microbiota of children diagnosed with pulmonary tuberculosis (PTB).

## Methods

### Study design

The design of the present study was a case-controlled, two-part study. Fecal samples were collected from PTB pediatric patients and healthy controls on the day of their admission. One-month after anti-tuberculosis treatment, a second analysis was performed to investigate alterations of the gut microbiota.

### Patients and information

A total of 18 patients with PTB were included in this study from the pediatric department of West China Second University Hospital, Sichuan University in China between June 2016 and June 2017. The diagnosis of PTB was identified according to the tuberculosis diagnosis and treatment guidelines for children in China [18]. Briefly, the diagnostic criteria of PTB was made by a combination of the following: A. pathogenic or pathological evidence including a smear or culture test positive for *Mycobacterium tuberculosis* in sputum, gastric juice or bronchoalveolar lavage fluid. B. Typical clinical symptoms of PTB including cough and fever lasting for more than 2 weeks and chest X-ray presenting typical signs of PTB; C. A history of contact with active TB patients or D. a positive tuberculin test (reaction diameter of the skin > 5 mm). E. Effective anti-tuberculosis treatment or F. the exclusion of other lung diseases, such as pneumonia, lung cancer, pulmonary cyst and interstitial lung disease were also considered. PTB was confirmed in some patients through a satisfaction of both A and B criteria. Other patients who were satisfied by a combination of B criterion and any two of C, D, E, or F criteria were diagnosed as probable PTB.

The same number of healthy controls were selected from children who completed regular physical check-ups at the hospital during the study period. All of the controls were free of any respiratory diseases, without a history of contact with active TB patients and had received Bacillus Calmette-Guerin vaccine. Finally, all control subjects had tested negative for tuberculin prior to enrollment.

PTB patients and healthy controls were matched for age, weight and sex. None of the enrolled participants had received probiotics, prebiotics or antibiotics one month prior to hospital admission/evaluation. Basic demographic and clinical data were collected at the time of initial hospital evaluation for all subjects. In addition, a follow up was completed for PTB patients after one-month initiation of anti-tuberculosis therapy.

Informed consent was signed by the guardians of each participant prior to sample collection. The protocol of the present study was approved by the Ethical Committee of West China Second University Hospital, Sichuan University.

### Sample collection and DNA extraction

Stool samples were obtained from healthy controls on the day of their hospital examination. For PTB patients, fecal samples were collected on the day of their admission and subsequently after a one-month anti-tuberculosis treatment. All samples were immediately frozen after collection and stored at − 80 °C. The genomic DNA was extracted from each sample using the E.Z.N.A.®Stool DNA Kit (D4015, Omega, Inc., USA).

#### Amplification and sequencing of 16S rRNA encoding gene

The bacterial 16S rRNA gene was amplified with forward primer 338F (5′-ACTCCTACGGGAGGCAGCAG-3′) and reverse primer 806R (5′-GGACTACHVGGGTWTCTAAT-3′). Sequencing was completed on Illumina Miseq platform (Illumina Inc., San Diego, CA, USA) and 300 bp paired-end reads were generated.

### Bioinformatic analysis

QIIME software, version 1.9.1 [19] (http://qiime.org/1.9.1) was used to complete the quality filtering of the readers as well as their taxonomic classification. The quality filtered readers were clustered into operational taxonomic unit (OTU) at 97% similarity threshold and the taxonomic assignment was performed with Greengenes database (Release 13.8, http://greengenes.secondgenome.com). Alpha diversity including the richness and diversity of bacteria in samples was measured by Chao 1 index, ACE index, Shannon index and Simpson index. The Chao 1 index and ACE index which reflect the amount of the OTUs of the microbiota were used to evaluate the richness of the community of gut microbiota. The diversity of microbiota was measured by Shannon index and Simpson index, which calculates the number of species and their relative abundance in the gut. Based on the the biological evolution information of sequences from each sample, the weighted Unifrac metric principal coordinates analysis (PCoA) was used to estimate the Beta diversity of the gut microbiota, which reflects differences of the gut microbiota community between groups.

### Statistical analysis

For the ratio of male to female, the Fisher’s exact test was completed. For age and weight, Chao 1 index, ACE index, Shannon index and Simpson index, comparison were performed by Mann-Whitney U test. The significant difference of Beta diversity was tested with Adonis non-parametric test. The relative abundance of gut microbiota between groups was compared by the T-test. *P*-values less than 0.05 were considered statistically significant. Fisher’s exact test and the Mann-Whitney U tests were performed by SPSS software, version 22.0 (IBM Corp, New York, NY, USA). The Adonis non-parametric test and T-tests were performed by R software, version 3.4.1 (https://www.r-project.org).

## Results

### Demographic data

During the study period, a total of 36 participants including 18 PTB patients (two confirmed cases of PTB and 16 probable PTB patients) and 18 healthy controls were enrolled. The age, sex and weight of participants in the two groups were matched (Table 1).

**Table 1:** Demographic characteristics of study subjects

	Patients with PTB (*n* = 18)	Healthy controls (*n* = 18)	*P* value
Male (%)	14 (77.8)	11 (61.1)	0.471
Age (year)	6.0 (0.2–15.5)	5.5 (0.6–16.0)	0.849
Weight (Kg)	19 (5–67)	22 (9–63)	0.200

### Diversity of gut microbiota in PTB patients and healthy controls

A total of 36 fecal samples were obtained from PTB patients and healthy controls on the day of their admission/hospital evaluation. About 1,044,454 sequencing tags were processed from the 18 PTB patients’ fecal samples, corresponding to 592 ± 259 OTU per sample. Another 18 samples from control subjects yielded 1,126,447 sequencing tags, corresponding to 716 ± 235 OTU per sample.

In the present study, the difference of microbiotal richness between the PTB group and healthy participants demonstrated no statistically significant difference. Although the Shannon index indicated no significant difference in gut microbiotal diversity between the two groups, a decreased Simpson index reflected that the diversity of fecal microbiota in PTB patients was significant lower than in healthy children (0.80 ± 0.20 VS 0.93 ± 0.04, p<0.05) (Table 2).

**Table 2:** Diversity of gut microbiota in patients with PTB and healthy controls

	Patients with PTB (*n* = 18)	Healthy controls (*n* = 18)	*P* value
ACE	780 ± 330	904 ± 252	0.20
Chao 1	791 ± 330	931 ± 241	0.16
Shannon	4.4 ± 1.5	5.4 ± 0.9	0.06
Simpson	0.80 ± 0.20	0.93 ± 0.04	0.04

### Community of gut microbiota in PTB patients and healthy controls

**Fig. 1:**
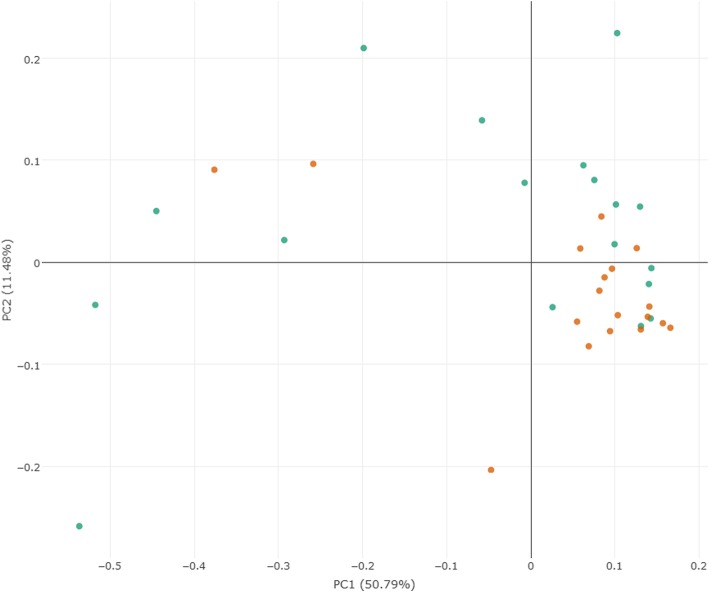
Principal coordinates analysis plots of PTB patients and healthy controls. The plots were based on weighted UniFrac distances. Green points represent PTB patients, orange points represent healthy controls

A weighted UniFrac PCoA was performed to identify the difference in fecal microbiota composition between PTB patients and healthy controls. As shown in Fig. 1, there was no significant differential clustering observed between samples from the two groups (R^2^ = 0.08, *P* = 0.08). This demonstrated that the total community of gut microbiota in the two groups exhibited no significant differences.

**Fig. 2:**
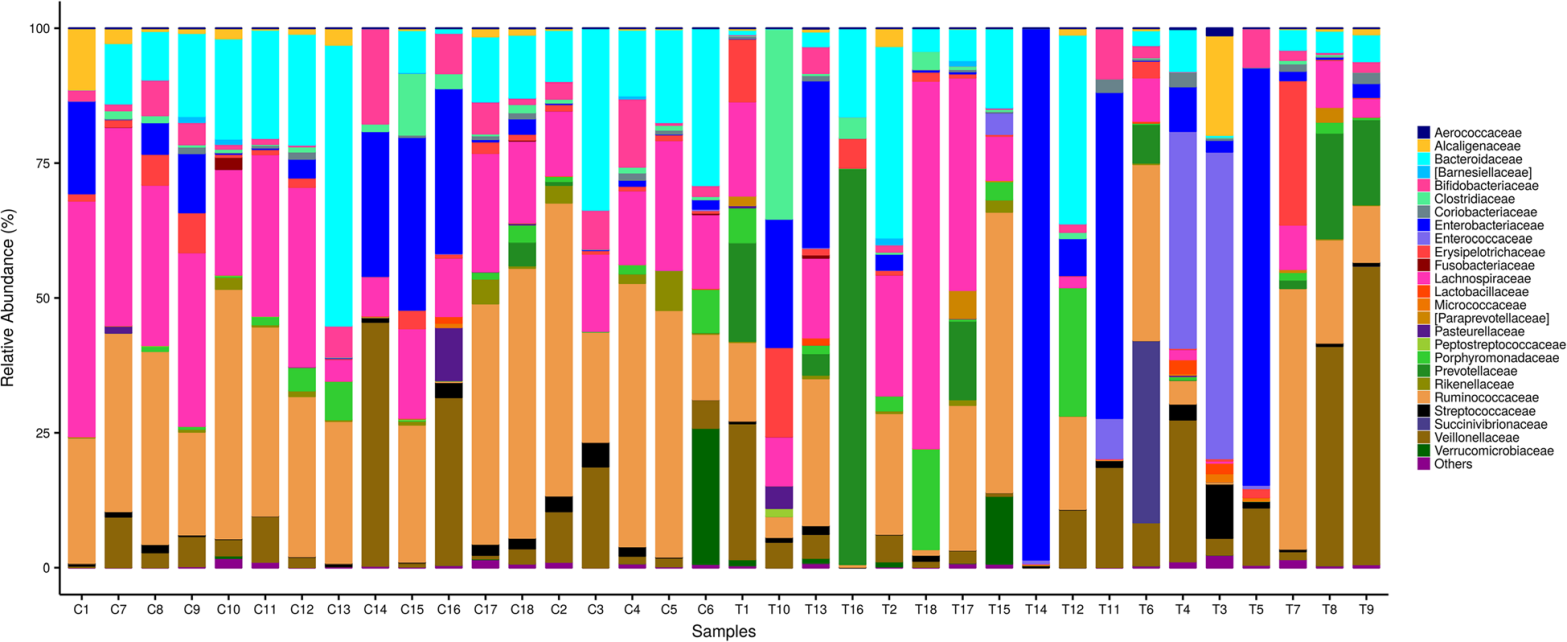
Comparison of relative abundance of gut microbiota at family level. Levels between the patients with PTB and healthy controls. T represents PTB group and C represents healthy controls. Bacteria with relative abundance of less than 1% in all samples are named as “others”. The bacteria names with square brackets are contested names due to polyphyly of the bacteria. [Barnesiellaceae], [Paraprevotellaceae] belong to Bacteroidetes phylum, Bacteroidia class, and Bacteroidales order

Although the whole community structure of gut microbiota in the two groups showed no meaningful differences, the relative abundance of fecal microbiota in PTB patients was distinct from healthy controls at the family, genus and species levels. According to the sequencing analysis, the community of gut microbiota in the PTB and healthy control groups consisted of four bacterial phyla: Firmicutes, Bacteroidetes, Proteobacteria, and Actinobacteria. The relative abundance of Firmicutes was 54.54 and 65.08% in the PTB and healthy subjects, respectively. Bacteroidetes, Proteobacteria, and Actinobacteria accounted for 20.72, 21.12 and 2.66% in PTB patients and 18.69, 9.62 and 4.92% of the gut microbiota in healthy controls. While there were no differences in the relative abundance of these four phyla between groups, at the family level (Fig. 2), when compared with healthy controls, PTB patients presented a significant abundance of *Enterococcaceae* (6.11% VS 0.02%, P<0.01) and *Prevotellaceae* (8.60% VS 0.29%, P<0.01) over healthy controls. A notable reduction of *Rikenellaceae* (0.30% VS 1.29%, P<0.05), *Bifidobacteriaceae* (1.75% VS 4.45%, P<0.05), *Lachnospiraceae* (11.80% VS 21.05%, P<0.05) and *Ruminococcaceae* (15.63% VS 30.60%, P<0.05) was also detected in the PTB group.

**Fig. 3:**
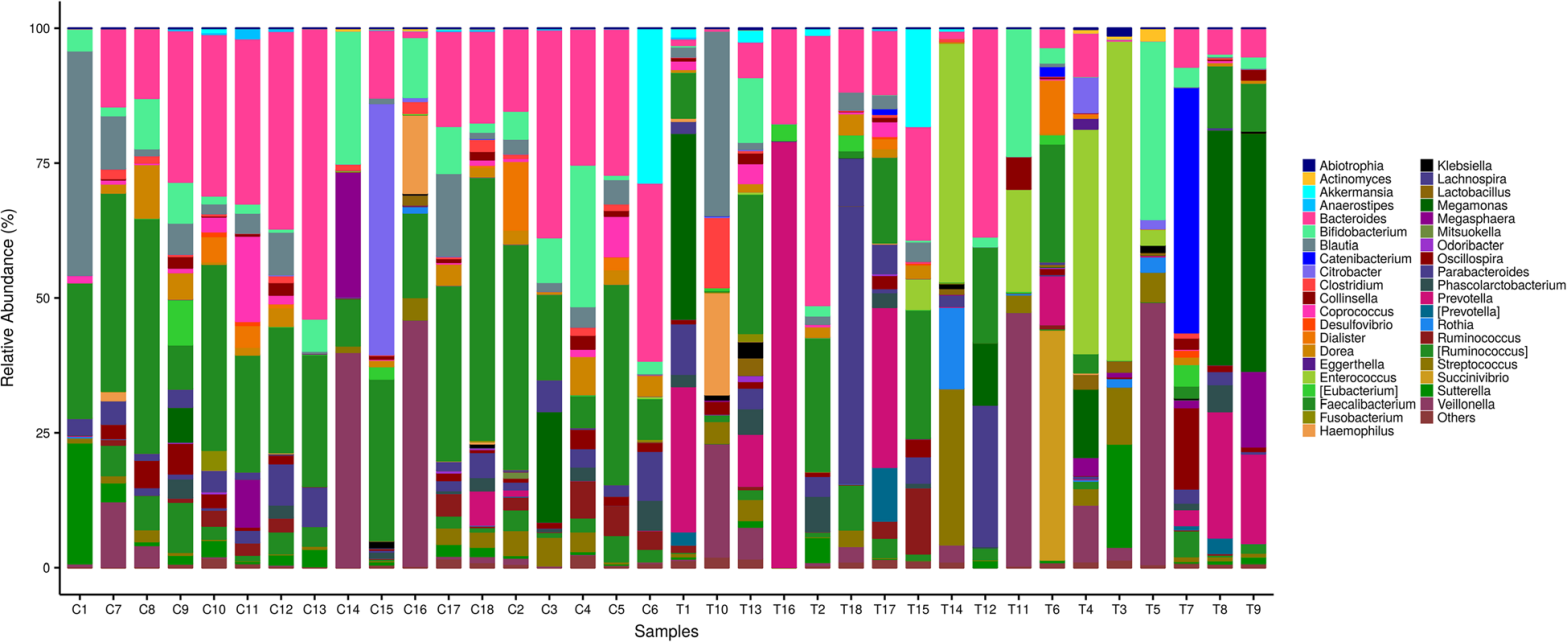
Comparison of relative abundance of gut microbiota at genus level. Levels between the patients with PTB and healthy subjects. T represents PTB group and C represents healthy controls. Bacteria with relative abundance of less than 1% in all samples are named as “others”. The bacteria names with square brackets are contested names due to polyphyly of the bacteria. [Eubacterium] belongs to Firmicutes phylum, Erysipelotrichi class, Erysipelotrichales order, and Erysipelotrichaceae family. [Prevotella] belongs to Bacteroidetes phylum, Bacteroidia class, Bacteroidales order, and [Paraprevotellaceae] family. [Ruminococcus] belongs to Firmicutes phylum, Clostridia class, Clostridiales order, and Lachnospiraceae family

At the genus level (Fig. 3), *Enterococcus* (9.61% VS 0.03%, P<0.01) and *Prevotella* (11.00% VS 0.44%, P<0.01) were found significant enriched in the PTB group when compared with healthy controls. On the other hand, the proportion of *Faecalibacterium* (9.23% VS 24.25%, P<0.01), *Bacteroides* (10.54% VS 21.90%, P<0.05), *[Ruminococcus]* (1.55% VS 4.16%, P<0.05) and *Dorea* (0.85% VS 2.52%, P<0.05) were significantly decreased in PTB patients versus controls.

**Fig. 4:**
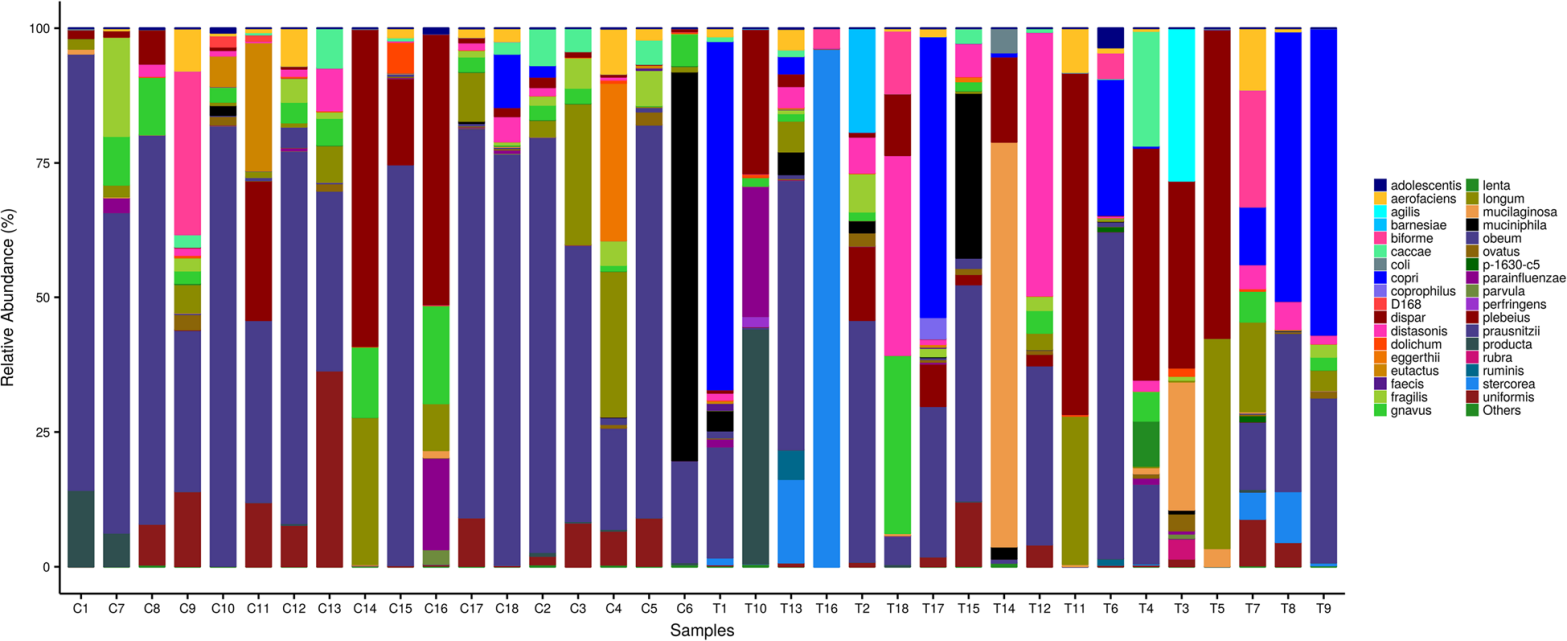
Comparison of relative abundance of gut microbiota at species level. Levels between the patients with PTB and healthy subjects. T represents PTB group and C represents healthy controls. Bacteria with relative abundance of less than 1% in all samples are named as “others”

At the species level (Fig. 4), results demonstrated that PTB patients tended to be abundant with *agilis* (1.57% VS 0, P<0.01), *barnesiae* (1.07% VS 0, P<0.01), *stercorea* (7.12% VS 0, P<0.01) and *copri* (14.68% VS 0.67%, P<0.01), while *prausnitzii* (20.61% VS 51.25%, P<0.01) was underrepresented compared with healthy subjects.

### Changes in gut microbiota of PTB patients before and after anti-tuberculosis treatment

Among the 18 PTB patients enrolled in our study, only 6 PTB patients (two confirmed cases of PTB and four probable PTB patients) completed the follow-up and provided fecal samples after receiving one-month of anti-tuberculosis treatment. These 6 patients were treated with a four-drug anti-tuberculosis regimen including isoniazid, rifampicin, pyrazinamide and ethambutol. After one-month of treatment, the clinical symptoms (such as cough, night sweating or fever) of these patients improved. This indicated the effectiveness of the anti-tuberculosis intervention.

A comparison of the microbiota obtained from fecal samples of these 6 PTB patients before and after one-month anti-tuberculosis treatment was carried out. The Chao 1 index (387 ± 103 VS 751 ± 299, P<0.05) and ACE index (388 ± 103 VS 739 ± 304, P<0.05) which reflect the richness of gut microbiota demonstrated a significant decrease after one-month of treatment. On the other hand, no significant difference was found though Shannon and Simpson indices which present the diversity of the microbiota (Table 3).

**Table 3:** Diversity of gut microbiota in PTB patients before and after one-month anti-tuberculosis treatment

	Patients before treatment (*n* = 6)	Patients post treatment (*n* = 6)	*P* value
ACE	739 ± 304	388 ± 103	0.03
Chao 1	751 ± 299	387 ± 103	0.03
Shannon	4.4 ± 1.3	4.8 ± 0.7	0.87
Simpson	0.82 ± 0.16	0.89 ± 0.06	0.75

**Fig. 5:**
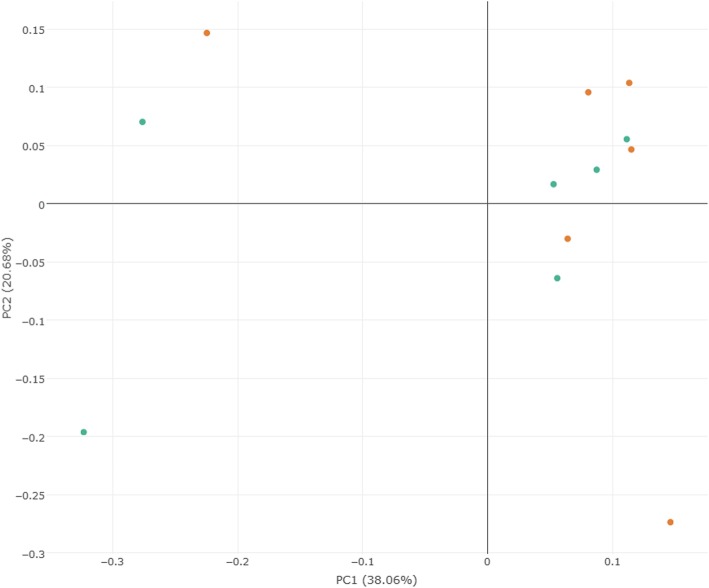
Principal coordinates analysis plots of 6 PTB patients before and after anti-tuberculosis treatment. The plots were based on weighted UniFrac distances. Green plots represent patients after receiving treatment, orange plots represent patients before treatment

In addition, the results of a weighted UniFrac PCoA demonstrated that the microbiota community as a whole (as represented from fecal samples of the 6 patients) could not be separated significantly (R^2^ = 0.08, *P* = 0.50) (Fig. 5). This indicated that the whole community of gut microbiota of PTB patients presented no obvious changes in gut microbiota after one month of treatment. Additionally, the relative abundance of the bacteria in these patients presented no significant changes after one-month of treatment.

## Discussion

The present study is the first to characterize the gut microbiota in pediatric PTB patients through the amplification of next-generation sequencing technology. The results of this study demonstrated that the patterns of gut microbiota in PTB patients were significantly different from healthy controls. The dysbiosis of gut microbiota in PTB patients was specifically characterized by an up-regulation of the pro-inflammatory bacteria *Prevotella* and the opportunistic pathogen *Enterococcus.* Further, a reduction of the beneficial bacteria *Ruminococcaceae*, *Bifidobacteriaceae* and *prausnitzii* was observed. This study also revealed that after one-month of effective anti-tuberculosis therapy, the richness of fecal microbiota is similarly decreased.

It is evident that the richness and diversity of gut microbiota plays a critical role in maintaining the health of the human body and that decreases in the richness and diversity of gut microbiota has been linked to diseases including influenza, asthma and HIV infection [13, 20, 21]. The results of this study indicated that the diversity of gut microbiota in PTB patients is generally decreased, which was demonstrated by the descending Simpson index value. This was not surprising given that some animal experiments have revealed similar findings [22, 23]. Winglee *et. al*. reported that mice infected with aerosolized *Mycobacterium tuberculosis* presented a rapid decrease in fecal microbiotal diversity [22]. In addition, Namasivayam *et. al.* found that compared with healthy controls, the diversity of gut microbiota in a murine model of TB presented a slight but significant reduction 12 weeks after infection [23]. In contrast, the results of Luo *et. al*. demonstrated that both new and recurrent TB patients maintained an increased richness of gut microbiota when compared with healthy subjects, and there were no significant differences in the Shannon and Simpson indices of fecal microbiotal diversity [24]. The discrepancies between that study and the present study may be due to differences in the subjects recruited. In the study by Luo *et. al.*, the participants were adults, some of which had previously received antibiotic treatment (prior to admission). However, the present study was focused specifically on the pediatric population. Additionally, none of the enrolled participants including the PTB group nor healthy controls had received antibiotics of any kind one month prior to hospital admission. We speculate that in either humans or mice, when infected with PTB, the local immunity of the lung is not singularly affected, rather the immune status of the intestine can be modulated due to crosstalk via the gut-lung axis. Thus, the altered immunity of the intestinal mucosa may contribute to a reduction of diversity in gut microbiota.

In the present study, although the total community of gut microbiota in patients with PTB and healthy controls presented no obvious differences, the relative abundance of some special bacteria at the family, genus and species levels were markedly different between the two groups. These results reflected a significant overrepresentation of *Prevotella* in PTB patients when compared with healthy controls. Previously, the increased abundance of *Prevotella* at mucosal sites has been reportedly involved with the development of many inflammatory diseases through the stimulation of the local and systemic immune response [25]. Similarly, Mutlu *et. al*. and Ling *et. al.* have reported that the relative abundance of *Prevotella* was predominant in HIV patients’ intestines when compared with healthy controls [26, 27]. Interestingly, a prospective exploration performed by Luo *et. al.* reported that the levels of *Prevotella* are positively correlated with the number of peripheral CD 4^+^ cells in new tuberculosis patients. They hypothesized that *Prevotella* may regulate the immune status of the host and therefore may be related to the prognosis and outcome of TB patients [24]. However, the exact mechanism of *Prevotella’s* role in the development of PTB in children remains unclear. Based on the findings of this study, we hypothesize that the increased abundance of *Prevotella* may activate pulmonary and systematic inflammatory reactions within the host to aggravate TB. This may be achieved through the regulation of the intestinal mucosa immunity and the subsequent increased production of pro-inflammatory cytokines, though this remains to be substantiated in a pediatric population.

*Enterococcus* is an opportunistic pathogen which usually inhabits the alimentary tract of humans and is associated with many infectious disease including urinary tract infections, surgical wound infections, and bacteraemia [28]. In this study, PTB patients demonstrated a significant abundance of *Enterococcaceae* and *Enterococcus* in comparison with the healthy controls. The research performed by Krisna *et. al*. presented a similar result, in that *Enterococcus* was more predominant in the TB sputum samples than healthy subjects [29]. Additionally, Sabino *et. al*. reported that the microbiota of patients with primary sclerosing cholangitis was characterized by an abundance of *Enterococcus* and that genus was associated with disease severity [30]. On the one hand, the predominance of *Enterococcus* in PTB patients was hypothesized to be associated with an impaired epithelial barrier and intestinal permeability, resulting in bacterial translocation from the intestinal tract into systemic circulation where an immune inflammatory reaction could be triggered, thus contributing to the development of tuberculosis. On the other hand, it is worth highlighting that some pathogens are species-specific. Thus, accurate identification of specific pathogen species presents a future direction of study.

Interestingly, the structural imbalance of gut microbiota in patients with PTB is not only coupled with significant overrepresentation of pro-inflammatory bacteria and opportunistic pathogens, but also the significant depletion of beneficial bacteria. In the present study, F. *ruminococcaceae* and F. *prausnitzii,* belonging to the genus *Faecalibacterium,* were significant lower in the PTB group than in healthy controls. Previous studies have described that F. *ruminococcaceae* and F. *prausnitzii* may exert beneficial effects on human health though their metabolites, short-chain fatty acids (SCFA) [31, 32]. SCFAs are able to affect lipid, glucose, and cholesterol metabolism in many tissues, where a large proportion are used as a source of energy [33]. SCFAs can also regulate the proliferation of colonic epithelial cells and enhance the permeability of the intestinal mucosa [34]. SCFAs, especially butyrate, seem to exert a profound impact on the maintenance of immune homeostasis through broad anti-inflammatory effects, such as the regulation, migration, and adhesion of immune cells, the expression of inflammatory cytokines, and inhibition of histone deacetylases. Cellular proliferation, activation and apoptosis via a host of other signalling pathways have also been implicated in the scope of SCFA’s effects [35]. Besides the significant depletion of butyrate-producing bacteria, another beneficial bacterium, *Bifidobacteriaceae,* was also found to be reduced in PTB group. As a common bacterium in the human gastrointestinal tract, *Bifidobacterium* (which belongs to the family *Bifidobacteriaceae*) exerts a lot of beneficial effects on the host [36, 37]. Some studies have reported that reductions of *Bifidobacterium* has been associated with many diseases, such as influenza, asthma and cystic fibrosis [13, 14, 38]. We hypothesize that the depletion of these beneficial bacteria promotes the development of the PTB through many pathways, including but not limited to reducing the immune response against the invasion of foreign microbes and inducing the dysbiosis of systematic inflammatory regulation.

The present study also elucidated a significant reduction of the richness of gut microbiota in PTB patients after one-month of anti-tuberculosis treatment. This finding is supported by research performed by Namasivayam *et. al* [23], which achieved similar results in mice after anti-tuberculosis treatment, wherein the richness of gut microbiota of the mice was similarly decreased. However, the relative abundance of gut microbiota between the patients upon admission and after one-month of treatment presented no significant difference in our study, which was in contrast to the results of Namasivayam *et. al* [23]. Namasivayam *et. al*. reported that the relative abundance of the gut microbiota in TB mice before and after anti-tuberculosis treatment were significantly different. Several factors may account for this discrepancy. One obvious difference would be the species of the subject population. While Namasivayam *et. al.* utilized an animal model of TB, the present study evaluated human children with TB. Another possibility is that the anti-tuberculosis treatment regimen was different between the two studies. In our study, all patients received a 4-drug regimen including isoniazid, rifampicin, pyrazinamide and ethambutol. But in the study of Namasivayam *et,* all mice received a 3-drug regimen including isoniazid, rifampin, and pyrazinamide. We speculated that changes in the gut microbiota of PTB patients after treatment may be linked to the following factors. First, the direct effect of the anti-tuberculosis drugs on the gut microbiota. Second, the bidirectionality of gut-lung interactions could be such that when the immunity of the pulmonary condition is improved, the local immunity of intestine (as well as gut microbiota) may be proportionally improved through the influenced of the ‘gut-lung axis’ cross-talk.

There were several limitations in the present study. First, owing to the risk of radiation exposure to healthy children, this study did not obtain chest X-ray to exclude PTB in healthy controls. However, tuberculin test, medical history and a physical examination were used to eliminate the possibility of PTB in healthy controls. It has been reported that the sensitivity and specificity of the tuberculin test for screening TB is greater than 80% [39], thus it can be reliably used to detect TB in subject pools. The present study was limited by the cross-sectional research design, inhibiting the ability to detect causal relationships between the gut microbiota and the development of PTB. Another limitation is the relative small simple size. Because only 18 patients were enrolled in our study, so we didn’t analyze the characterization of gut microbiota in PTB patients in different age groups including infant, childhood and adolescent groups. What’s more, present study only explored changes in the gut microbiota of six PTB patients after one-month of anti-tuberculosis treatment and therefore may not comprehensively reflect the changes in the gut microbiota of pediatric PTB patients after anti-tuberculosis treatment. Finally, a large-scale, multi-center study would be required to validate this initial characterization of the gut microbiota of pediatric TB patients.

## Conclusions

The present study provided the first detailed analysis of the characterization of gut microbiota in children with PTB, including changes of the intestinal microbiota after one-month anti-tuberculosis treatment. Our results demonstrated that the gut microbiota in PTB patients was significantly different from healthy controls as characterized by the increase of pro-inflammatory bacteria and opportunistic pathogens and the reduction of beneficial bacteria. In addition, longitudinal profiling revealed a significant decreased richness of gut microbiota in PTB patients who received one-month anti-tuberculosis treatment. It was hypothesized that the above mentioned changes of the gut microbiota could affect the development of PTB through the downstream regulation of the immune status of the host by way of the gut-lung axis.

## Data Availability

Not applicable

## Abbreviations

OTU: Operational Taxonomic Unit
PCoA: Principal Coordinates Analysis
PTB: Pulmonary Tuberculosis
TB: Tuberculosis
